# COVID-19: The Cause of the Manifested Cardiovascular Complications During the Pandemic

**DOI:** 10.3389/fcvm.2021.744482

**Published:** 2021-10-28

**Authors:** Audditiya Bandopadhyay, Alok Kumar Singh, Gyaneshwer Chaubey

**Affiliations:** ^1^Cytogenetics Laboratory, Department of Zoology, Banaras Hindu University, Varanasi, India; ^2^M.D.D.M. (Cardiology), Senior Intervention Cardiologist, Lifeline Hospital, Varanasi, India

**Keywords:** SARS-CoV-2, inflammation, myocardial damage, heart, CVD

## Abstract

In the course of human history, we encountered several devastating waves of pandemics, affecting millions of lives globally and now the rapid and progressive spread of the novel SARS-CoV-2, causing Coronavirus disease (COVID-19) has created a worldwide wave of crisis. Profoundly straining national health care systems, it also significantly impacted the global economic stability. With the introduction of COVID-19 measures, mainly driven by immunization drives, casualties due to the virus were reported to decrease considerably. But then comes into play the post-Covid morbidities, along with their short and long-term effects on the elderly and the co-morbid population. Moreover, the pediatric population and the otherwise healthy cohort of the young athletes were also reported being largely affected by the varying amount of post-recovery virus-induced Cardiac manifestations, in the subsequent waves of the pandemic. Therefore, here we thrived to find answers to the seemingly unending series of questions that popped up with the advent of the disease, nevertheless, there still lies a blind spot in understanding the impacts of the disease on the Cardiovascular Health of an individual, even after the clinical recovery. Thus, along with the current data related to the diverse cardiovascular complications due to SARS-COV-2 infection, we suggest long-term ‘Cardiac surveillance' for the COVID-19 recovered individuals.

## Introduction

In late 2019, a cluster of cases of “pneumonia of unknown origin,” emerged, the epicenter of which was linked to the seafood wholesale market in Wuhan, China, that heralded the onset of Coronavirus disease (1). However, there are further reports suggesting that this virus was already circulating in China before the seafood market cluster event (https://www.sciencemag.org/news/2020/01/wuhan-seafood-market-may-not-be-source-novel-virus-spreading-globally). The disease spread rapidly to several countries around the globe and was already declared a pandemic by WHO. To date, a total of 187,086,096 confirmed cases of COVID−19 with a mortality of 4,042,921 have been reported (2). COVID−19 questioned the existence of mankind in the twenty-first century not just by crippling the global healthcare system but also contributing to the psychological and socio-economic burden on the entire humanity.

The family of seven known human Coronaviruses has long been associated with emerging respiratory distress syndromes and flu-like outbreaks. This is the reason behind the high occurrence of cases of pneumonia and bronchitis in patients with a severe COVID-19 infection. In the past two decades, two recorded epidemics were caused by the same family of the virus—Severe Acute Respiratory Syndrome Coronavirus (SARS-CoV), in 2002–2003, and more recently, the Middle East Respiratory Coronavirus (MERS-CoV) in 2012, has widely been mentioned. Previously known human coronavirus variants, which were associated with the common cold—HCoV-229E, HCoV-NL63, HCoV-OC43, and HCoV-HKU1, have not yet been found to be associated with heart abnormalities. But there are few reports of the patients suffering from the Middle East respiratory syndrome (MERS; caused by MERS-CoV) with myocarditis and a few cases of cardiac disease in the patients who suffered from SARS (caused by SARS-CoV) (3, 4). However, recent literature reported serious cardiovascular complications occurring in about 10–20% of hospitalized patients, apart from the respiratory effects of COVID-19; and the patients who suffered from pre-existing heart ailments may suffer either a heart attack or congestive heart failure (5). This deciphers distinct characteristics of SARS-CoV-2 in its comprehensive cardiac involvement, which could also be a consequence of the exposure of the virus to millions due to the pandemic. Reports also stated that COVID-19 triggered inflammation of the heart muscle—Myocarditis (6). The most recent severe acute respiratory syndrome coronavirus 2 (SARS-CoV-2), displayed tropism for the heart and can lead to myocarditis (inflammation of the heart), necrosis of its cells, mimicking heart attacks, arrhythmias, and acute or protracted heart failure (muscle dysfunction) (3). These complications, which at times are the sole features of COVID-19 clinical presentation, have occurred even in the cases with milder symptoms and in people who did not experience any symptoms. Unsuspected cardiac involvement including sudden cardiac death, in such healthy and young athlete groups, has further elevated the concerns regarding our current knowledge about the impact of the disease on heart health.

## Structural Aspects of COVID-19 Virus

**Figure 1:**
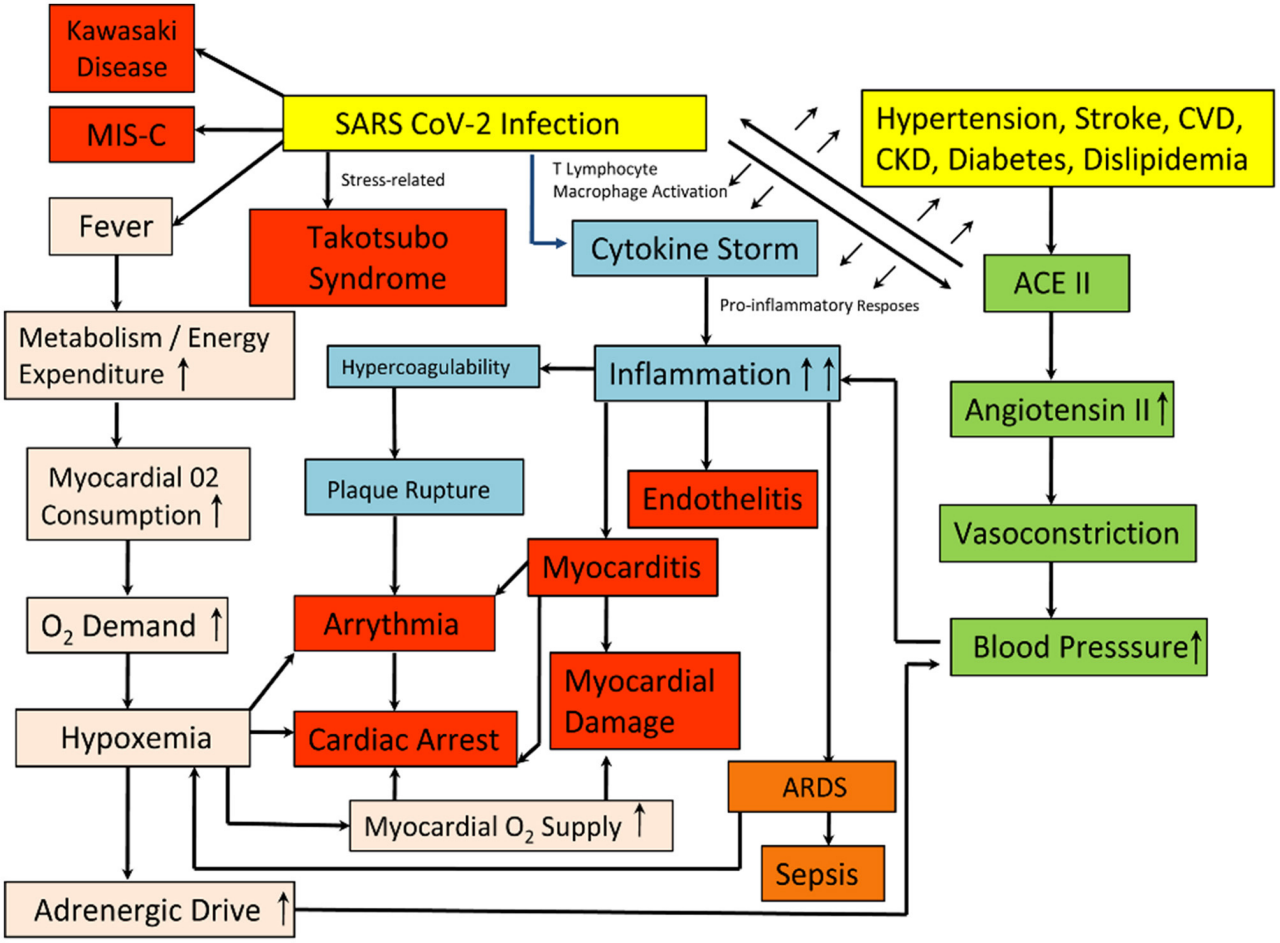
Schematic representation of the pathways leading to post-COVID Heart conditions. The Yellow boxes represent the causes, whereas the Red and the Orange boxes depict the consequences of the infection on the heart and the lungs. The Pink, Blue, and the Green boxes depict the various systemic pathways involved, leading to the development of the conditions.

The difference between SARS-CoV-2 and SARS is apparently a furin polybasic site that alters their structure, and when cleaved, broadens the types of cells (tropism) that the virus can infect (7). It is a large family of single positive-stranded, enveloped RNA virus that finds its host in several animals, and by methods not yet explained, they can pass from one species to another. The virus targets the angiotensin-converting enzyme 2 (ACE2) receptor throughout the body, which facilitates the entry of viral genetic material by the means of its spike protein, along with the assistance of the cellular serine protease transmembrane protease serine 2 (TMPRSS2), heparan sulfate, and other proteases, which cleaves the viral spikes protein and make the entry pathway for the viral genetic contents (8). So, the higher the ACE2 receptor's number of receptors in any cell, the higher the susceptibility for the viral entry and greater viral load possibility. The involvement of ACE2 in the regulation of blood volume, systemic vascular resistance, and thus cardiovascular homeostasis is monumental (Figure 1). Previous studies have shown its association with hypertension, stroke, dyslipidemia, and cardiovascular diseases, and kidney diseases (9–12). The heart also has a high level of ACE2 expression which makes it more susceptible to the SARS-COV-2 infection. The affinity of SARS-CoV-2to ACE2 is significantly higher than that of SARS (13), and thus it may perturb the angiotensin-renin pathway severely.

## COVID-19 and Its Systemic Implications

The tropism to other organs beyond the lungs has been quoted in some studies from the autopsy specimens. It was found that the SARS-CoV-2 genomic RNA was the highest in the lungs. However, in the heart, kidney, and liver, considerable amounts of viral load were detected in 16 out of 22 deceased patients (14). In a report from (15), out of a series of an autopsy of 39 deceased COVID-19 patients, only 31% had a high viral load, i.e., above 1,000 copies, in the heart while ~38% of the deceased was not found to possess a detectable viral load in the myocardium. Accordingly, SARS-CoV-2 infection can damage the heart in both direct and indirect ways. *In-vitro* studies have shown the ability of SARS-CoV-2 to infect the induced pluripotent stem cells (iPSCs) derived cardiomyocytes, causing the distinctive pattern of cell fragmentation along with the complete dissolution of the contractile machinery (16). In another iPSC study, SARS-CoV-2 infection leads to apoptosis, and ultimately the heartbeat ceases within 72 h of the viral exposure (17). Besides the direct involvement of the viral infection in the heart muscles, its entry into the endothelial lining of the blood vessels of the heart and multiple vesicular beds has also been reported. Another potential threat is the effects of secondary immune response in the infected heart and endothelial cells (endothelitis) which may include the dysregulation of the renin-angiotensin-aldosterone system modulating blood pressure; activation of pro-inflammatory responses including platelets, neutrophils, macrophages, and lymphocytes, the cytokine storm and a prothrombotic state (Figure 1).

There is a varying level of cardiovascular manifestations, oscillating from limited necrosis of cardiac cells leading to myocarditis to an often-fatal failure of the heart to pump sufficient blood leading to cardiogenic shock (18). One out of every five hospitalized COVID-19 patients suffering from cardiac injury reflects an accumulation of troponin (a cardiac muscle-specific marker) in blood and the same happens with those having pre-existing heart ailments. Also, for this kind of myocardial injury in-hospital mortality, troponin accumulation is an indicator of morbidity risk (19). Moreover, it has been observed that patients with higher troponin amounts also have increased levels of many inflammatory markers [including interleukin-6(IL-6), C- reactive protein, ferritin, lactate dehydrogenase (LDH), and an increased neutrophil count] and heart dysfunction (amino-terminal pro-B–type natriuretic peptide) (20). Conversely, an immunologic basis is likely as there is a possibility of myocarditis results from the hyperimmune response in order to tackle coronavirus by releasing excess cytokines. Cytokines could result in inflammation that damages the lungs and the heart alike. This condition, known as a cytokine storm, is more serious in the elderly and the co-morbid population. However, it was also seen to affect the middle-aged population largely during the subsequent waves of the pandemic in India. It is the primary reason for the severe respiratory complications which lead to death in patients suffering from coronavirus (21). Cytokines promote blood coagulation and thus, interfere with the body's clot-busting system. Blood clots in coronary arteries in turn can block blood flow and cause heart attacks. A tendency for clotting, both in the microvasculature and large vessels, has been reported in multiple autopsy reports and in young COVID-19 patients with a history of stroke. Another relevant possibility could be the development of cardiac complications in some coronavirus patients, as a consequence of infections in their lungs. Insufficient oxygen increases the risk of arrhythmias. At the same time, fever caused by the virus increases the body's metabolism, thus the cardiac output. As a result, the patient's heart struggles with an elevated oxygen demand along with a reduced supply, causing an imbalance that leads to a myocardial injury. The causes of death might involve multiple organ dysfunctions in most cases, and therefore it is difficult to differentiate the myocardial injury as the sole reason for such cases. Schematic representation of the different pathways leading to the post-COVID conditions has been depicted in Figure 1.

## Cardiovascular Health with the Rise of COVID-19

With an ascent in the number of COVID-19 confirmed cases and the accumulating clinical data, in addition to the common presentation of respiratory failure, the cardiovascular manifestations induced by this viral infection have generated considerable concern (22). Huang et al. (23) reported that 12% of the patients with COVID-19 were diagnosed to have an acute myocardial injury, manifested due to the elevated levels of high-sensitive troponin I. This was further supported by reports stating 16.7% out of 138 hospitalized patients with COVID-19 had suffered from arrhythmias and 7.2% had an acute myocardial injury (24). However, the plausible cause of the COVID-19 infection in the development of myocardial injury in the hospitalized patients suffering from underlying cardiovascular disease (CVD) is still unknown and it requires extensive study. Although still unclear, whether it is an after-effect of a hyperactive immune response against the virus or the virus itself leading to myocardial inflammation which is associated with cardiac function impairment and ventricular tachyarrhythmias.

Myocarditis is a diffuse pattern of inflammation of the heart, typically representing a variable admixture of injury and an inflammatory response to the injury, and may extend through all the three layers of the human heart to the pericardium, encompassing the heart. This is even more worrisome than the restricted pattern injury. The immunological and the inflammatory response is one of the most common observations at the autopsy studies after SARS-CoV-2 infections, unlike the SARS-associated myocarditis, which didn't show any lymphocyte infiltration. Conduction block and malignant ventricular arrhythmias, both of which can lead to cardiac arrest, can occur when myocytes, which synchronize electrical conduction, are involved. Besides in-hospital arrhythmias, numerous geographic regions with high COVID-19 dissemination have been reported to observe a steep increase in out-of-hospital cardiac arrest and sudden death. There has been a rise of 77% in the cases in Lombardy, Italy, as compared to the previous year (25). Due to a cluster of chest pain-like sensations, an irregular EKG, and high levels of cardiac-specific enzymes in the blood, myocarditis imitating a heart attack has been reported in individuals as young as 16 years old (3). Heart failure, acute cor pulmonale (right heart failure with potential pulmonary emboli), and cardiogenic shock can occur when there is significant and diffuse heart muscle injury. Other pathways that could also be responsible for COVID-19-related heart dysfunction, such as Takotsubo syndrome or the Broken heart syndrome (a transient stress-related illness that causes apical ballooning), ischemia caused by endocarditis, and related atherosclerotic plaque rupture with thrombosis were reported (3). Other causes included the multisystem inflammatory syndrome of children (MIS-C), although MIS-C reported here, was not only exclusive to children but also the same clinical features have been the subject of case reports in adults, such as in a 45-year-old (3).

Although the children were thought to be less susceptible to COVID-19, as compared to the adults, and while the majority of them with COVID-19 were asymptomatic or presented with only milder forms of the symptoms, the reports of COVID-19 associated severe inflammatory symptoms among the pediatric patients were not null (26–28). An unexpected cluster of eight children (aged 4–14 years) presenting with a hyperinflammatory syndrome with symptoms of Kawasaki Disease was reported in a case series from the United Kingdom (26). COVID-19 patients who underwent magnetic resonance imaging (MRI) or echocardiogram of the heart have recently revealed some fresh information concerning some cardiac involvement (29–31). In one such study, the left and the right ventricular abnormalities were reported in 479 out of 1,216 patients, and 397 patients, respectively, with evidence of new myocardial infarction in 36 of them. Myocarditis was reported in 35 and Takotsubo Cardiomyopathy in 19 patients in the same study. Severe cardiac disease (severe ventricular dysfunction or tamponade) was also observed in 15% of the patients. And in those without any pre-existing cardiac disease, the echocardiogram was abnormal in 46%, and 13% of the cases had severe disease. Patients were between 52 and 78 years old (30). In another study, 15 patients had abnormal CMR findings on conventional CMR sequences: myocardial edema was found in 54% of patients, and LGE was found in 31% of the patients reduced right ventricle performance which includes ejection fraction, cardiac index, and stroke volume per body surface area were found in patients with positive conventional CMR findings (31). A group of 100 individuals recovered from the illness, but 78 had cardiac abnormalities, including 12 of 18 patients who had no symptoms, and 60 showed continuing myocardial inflammation, which is consistent with myocarditis (29). These findings point to the necessity for more research on covid-19's long-term cardiovascular effects. The majority of over 1,200 individuals with COVID-19 in a large prospective cohort had echocardiographic abnormalities (30). This raises questions about whether heart involvement is considerably more common than previously thought, especially because at least 30–40% of SARS-CoV-2 infections are asymptomatic. Because all of these patients did not have a systematic cardiovascular assessment for any probable myocarditis or other heart abnormalities, which could explain some of the lingering symptoms, they may have hidden underlying cardiac pathology.

## Role of Angiotensin-Converting Enzyme and It's Inhibitors in COVID-19

Angiotensin-converting enzyme inhibitors (ACEIs) and angiotensin receptor blockers (ARBs), both of which are known to block the renin-angiotensin system (RAS), also might affect an individual's susceptibility to COVID-19 and further worsen its severity (32–35). Angiotensin II, the main effector molecule in the renin-angiotensin-aldosterone system (RAAS), is upregulated in many clinical conditions, for which inhibition of angiotensin II by RAAS inhibitors is a common therapeutic strategy. Angiotensin-converting enzyme (ACE) produces angiotensin II from angiotensin I, whereas ACE2 inactivates angiotensin II by converting it to angiotensin (1–7) (34). Therefore, ACE2 has been assumed to have a protective effect against cardiovascular disease and lung injury. It has been shown that the RAAS inhibitors may increase the ACE2 expression, thus raise concern among COVID-19 positive patients (33).

On the other hand, a study reported significant interactions between ethnicity and ACE inhibitors and ARBs for COVID-19 disease. The risk of COVID-19 disease associated with ACE inhibitors was shown to be higher in the Caribbean and Black African groups than the white group. Variations among the ethnic groups raise the possibility of ethnic-specific effects of ACE inhibitors/ARBs on COVID-19 disease susceptibility and severity (36). Another study found that the administration of ACEI/ARB drugs had a positive effect on reducing D-dimer and the number of people with fever (37). As a result of such paradoxical issues of using ACEIs/ARBs during COVID-19, it is still an area requiring extended investigation to prove. However, in the setting of coronavirus disease, downregulation of ACE2 by severe acute respiratory syndrome coronavirus 2 (SARS-CoV-2) infection might be involved in mediating cardiovascular damage, besides, the medications that have been proposed as treatments for COVID-19 such as hydroxychloroquine and azithromycin have pro-arrhythmic effects, AF, atrial fibrillation; VF, ventricular fibrillation; VT, ventricular tachycardia (38).

## Present Scenario

Previous studies have depicted the overall clinical attributes and epidemiological findings of patients with COVID-19, and a portion of it has shown that the condition of some patients with COVID-19 deteriorates rapidly. In contrast to the asymptomatic, a substantial proportion of people suffer a long-standing, often incapacitating illness, called long-COVID. Typical symptoms of this include fatigue, difficulty in breathing, chest pain, and abnormal heart rhythm (39, 40). While the patients with underlying Cardiovascular pathology, but without myocardial injury put up with a relatively favorable prognosis, myocardial injury is much more common in patients with COVID-19 and has been found to have a significant association with the fatalities due to COVID-19.

The most intriguing question that stirred up in this while is that why do certain individuals have a propensity for heart involvement after the SARS-CoV-2 infection? Studies deciphered that the infected patients who get myocarditis do not necessarily have any more virus in their bodies than those who do not foster the condition. The prediction once recognized a few months into the pandemic, was that the cardiac involvement would chiefly occur in patients with severe COVID-19. Clearly, it is found to be much more common than anticipated. However, the true incidence is unknown. Primarily, it is vital to determine any cause that drives the pathogenesis. Whether it represents an individual's inflammatory response, an autoimmune phenomenon or some other explanations are yet to be clarified.

Beyond the prevention of COVID-19 infections, the goal of averting cardiovascular involvement is paramount. The marked heterogeneity of the disease, ranging from lack of symptoms to fatality, is poorly understood. A newly emerged virus, widely circulating throughout the human population, with a panoply of manifestations, has made this especially daunting to untangle. It wouldn't surprise much in the future if the patients present with cardiomyopathy of unknown etiology and test positive for SARS-CoV-2 antibodies. However, attributing all such cardiomyopathy solely to the virus may be difficult, given the high prevalence of infections. A biopsy might be a necessity to identify any virus particles to support any causality.

These sudden after-effects could be attributed and studied to be validated at two different levels. First is the entry point for the virus, that is the ACE2 receptors and their variations among the individuals of certain ethnicities, which makes them more susceptible or resistant toward the virus. There have been studies concluding the polymorphism within the ACE2 gene within the populations that explains the outcome, on comparing the Western and the Indian populations, and their affinity with the East Asians (41).

The other points to be considered include the immunity of the individual and its effects after the entry of the virus. The reaction of the immunity toward the non-self determines its activity, and thus results in a hyperactive state of immune responses, that leads to systemic inflammation, which prevails for a much longer time, as compared to the symptoms themselves. The classification of asymptomatic people for COVID-19 is vague. And in many cases, the asymptomatic individuals are sometimes just the result of the symptoms getting masked due to ignorance or the socio-economic background of those individuals. They may have underlying inflammation-related pneumonia due to the disease and still not experience any level of hypoxia and thus, be considered asymptomatic. On the other hand, the body of the athletes, in practice, may demand more oxygen and experience the symptoms of hypoxia and thus, can lead to cardiac arrest due to pulmonary thromboembolism, as a consequence of dilated arteries due to the disease-related inflammation (42). The same demographic group of young and healthy, that is most common to lack the symptoms after SARS-CoV-2 infections, raises the question of how many athletes have an occult cardiac disease. Systematic assessment through some form of cardiac imaging and arrhythmia screening of athletes, who test positive for SARS-CoV-2, irrespective of symptoms, seems prudent until more is perceived. The authors in a study reported on a cohort, consisting of a large sample size of 2,461 athletes, of whom 1,597 (64.9%) had the complete comprehensive screening testing, including CMR imaging without prior selection, where they found that 37 (2.3%) of these athletes demonstrated diagnostic criteria for myocarditis by CMR imaging, including 20 without cardiovascular symptoms and with normal ECG, echocardiography, and troponin test results, who would not have been identified without CMR imaging (43). However, another subsequent study was published, where a cohort of 145 competitive athletes, who had tested positive for COVID-19 with either mild to moderate or, no symptoms, were evaluated approximately 15 days post-positive test result, using cardiac MRI, EEG, and serum markers of cardiac pathology, and only two were found to have MRI findings consistent with myocarditis. This led to conclude that its incidence following COVID-19 was much less prevalent than previously thought (44). Controversies remain until the results are validated further, on larger cohorts, considering ethnicity (ancestry) as a parameter, as that could play a vital role in the risk prediction of an individual.

## Future Direction and Conclusion

Long-term observation and prospective study design (Cardiac Surveillance) on the viability of treatments, explicit for myocardial injury are of utmost significance. Further, aggressive treatment may be considered for patients with myocardial injury. Therefore, monitoring of myocardial injury markers and cardiac function is of extreme importance, and attention should be paid to the early identification and comprehensive management of myocardial injury in such patients. But what has so far driven populations to be more vulnerable to post-COVID morbidities?

We hypothesize it as the genetic variability among the individuals at these two tiers, making them more or less susceptible toward the mentioned long-standing ailments, which are probably more severe than the disease itself. There comes into play the role of genetic mapping. Genome-wide analysis (GWAS) and Whole-genome analysis (WGA) study designs would reveal and map a particular population at risk, would categorize the vulnerable groups to prioritize them at first, and thus manage the casualties due to the disease burden.

## Author Contributions

GC and AB conceived and designed the study. AB, GC, and AS constituted the manuscript. All authors contributed to the article and approved the submitted version.

## Funding

GC was supported by Faculty IOE Grant BHU (6031). AB was supported by UGC-RET and CAS fellowship. No funders had a role in study design, data collection and analysis, decision to publish or preparation of the manuscript.

## Conflict of Interest

The authors declare that the research was conducted in the absence of any commercial or financial relationships that could be construed as a potential conflict of interest.

## Publisher's Note

All claims expressed in this article are solely those of the authors and do not necessarily represent those of their affiliated organizations, or those of the publisher, the editors and the reviewers. Any product that may be evaluated in this article, or claim that may be made by its manufacturer, is not guaranteed or endorsed by the publisher.
